# Robot-assisted urological surgery in the Middle East: Where are we and how far can we go?

**DOI:** 10.1080/2090598X.2019.1601003

**Published:** 2019-04-23

**Authors:** Raed A. Azhar, Mohamed A. Elkoushy, Saad Aldousari

**Affiliations:** a Department of Urology, Faculty of Medicine, King Abdulaziz University, Jeddah, Saudi Arabia; b Department of Urology, Faculty of Medicine, Suez Canal University, Ismailia, Egypt; c Department of Surgery, Urology Unit, Faculty of Medicine, Kuwait University, Kuwait City, Kuwait

**Keywords:** Minimally invasive surgery, robot-assisted surgery, Middle East, radical prostatectomy, radical cystectomy, radical nephrectomy

## Abstract

**Objectives**: To evaluate robot-assisted surgery (RAS) in Urology in the Middle East, and its status and future perspectives.

**Methods**: A Medical Literature Analysis and Retrieval System Online (MEDLINE) search was performed using the following keywords: ‘robotics’, ‘robot-assisted surgery’, ‘laparoscopy’, at first with each specific procedure name, such as radical cystectomy, followed by ‘Middle East’ and country names. All abstracts and articles in English that adhered to the scope of the current issue were selected, giving special consideration to relevant landmark articles and those describing trends and the future of RAS in Urology.

**Results**: Only a few index case reports characterised RAS in the Middle East. The Middle East possess only 1% of the da Vinci® Surgical Systems (Intuitive Surgical Inc., Sunnyvale, CA, USA) installed worldwide, including 19 in Saudi Arabia; six in Qatar; two in each of Kuwait and Lebanon; three in the United Arab Emirates; and only one in Egypt. The total number of RAS performed in the Middle East is low compared to Europe and the USA. Many countries in the Middle East still lack surgical robots despite having the expertise and appropriate caseload, whilst others seem not to utilise the surgical robot at a suitable rate, as reflected by the sparse number of operated cases and outgoing publications. There are major differences in RAS availability, usage, and perception according to the geographical place of practice and acceptance of robots by surgeons and patients.

**Conclusion**: RAS in Urology continues to grow in the Middle East, with increasing caseloads and diversity of operated cases. Acceptance of robots by Middle East surgeons is significantly increasing.

**Abbreviations**: 3D: three-dimensional; KSA: Kingdom Saudi Arabia;MIS: minimally invasive surgery; RAA: robot-assisted adrenalectomy; RAP: robot-assisted pyeloplasty; (O)(RA)PN: (open) (robot-assisted) partial nephrectomy; RAS: robot-assisted surgery; (O)(RA)RC: (open) (robot-assisted) radical cystectomy; (RA)RP: (robot-assisted) radical prostatectomy; SAUC: Sabah Al-Ahmad Urology Center

## Introduction

Minimally invasive surgery (MIS) has the advantages of improved perioperative outcomes, shorter recovery, reduced blood loss and perioperative complications compared to open surgery [1]. Robot-assisted surgery (RAS), as an evolving worldwide MIS, has been successfully adopted rapidly over the last decade in Europe and USA in complex techniques, including oncological procedures involving the prostate, kidney and urinary bladder [2–4]. The emergence of robots, with three-dimensional (3D) magniﬁed vision, EndoWrist® (Intuitive Surgical Inc., Sunnyvale, CA, USA) technology, depth perception, and precision with intuitive movement, has made intracorporeal dissection and suturing easier. Robotic surgical systems interpose a computer between the surgeon’s hands and the tips of extremely small devices, with designed programs helping to perform all complex procedures through tiny ports. Moreover, surgical robots enable less experienced surgeons to perform MIS using the robot after a relatively short learning curve [5,6]. Nevertheless, widespread acceptance of robotics may be influenced by the lack of tactile feedback, fixed-port system, longer operative times, and cost [7,8].

According to Intuitive Surgical, the manufacturer of the da Vinci® Surgical System (Intuitive Surgical Inc.), the number of RAS has significantly increased in the last decade, with ~570 000 da Vinci procedures performed worldwide in 2014. By September 2017, there were 4271 systems installed worldwide, including 2770 (65%) in the USA, 719 (17%) in Europe, 561 (13%) in Asia, and 221 (5%) in the rest of the world. The installed base grew 13% (year-on-year) in the first quarter of 2018 to 4528 units. Most RAS data, programmes, and leaflets came from North America and Europe, where urologists with formal training perform most of the RAS procedures [9].

It seems that there is major diversity in RAS availability, usage, and conception, depending on the geographical place of practice. Unfortunately, the introduction and awareness of RAS in the Middle East are significantly lower than in Western countries. One of the major reasons why the robots are poorly utilised is the lack of expertise and the low volume of cases.

Currently, there are 44 robotic da Vinci Surgical Systems installed in the Middle East, including 13 active systems and six obsolete systems (standard and S systems) in Saudi Arabia (KSA), six in Qatar, three in the United Arab Emirates (UAE), and two in each of Kuwait and Lebanon. The availability of these robots has not translated into a better quantity or quality of case performance, with a lack of preferable marketing and non-recording of the operated cases.

## Methods

A Medical Literature Analysis and Retrieval System Online (MEDLINE) search was performed using the following keywords: ‘robotics’, ‘robot-assisted surgery’, ‘laparoscopy’, at first with each specific procedure name, such as radical cystectomy, radical prostatectomy, radical nephrectomy, partial nephrectomy, pyeloplasty, etc. Then, ‘Middle East’ and country names were added, such as Saudi Arabia, Egypt, Lebanon, United Arab Emirates, Kuwait, Qatar, Tunisia, Bahrain, and Oman. Abstracts and articles in English that adhered to the scope of the current issue were selected, giving special consideration to relevant landmark articles and those describing trends and the future of RAS in Urology. The number of da Vinci Surgical Systems installed globally was obtained from the official Intuitive Surgical site, available at the online 3-monthly presentation. Some described figures have been acquired from the operational records collected by the local distributor of the da Vinci Surgical System.

## Results

Only a few index case reports characterised the trends of RAS in the Middle East. A single article and a few case reports came from the KSA, one published article and two other abstracts came from Kuwait, three reports were published from Egypt, and a sparse data came from Lebanon, Qatar and the UAE, which did not go beyond online commercial data or social media. By September 2018, a total of 4814 da Vinci Surgical Systems were installed worldwide, including 3010 in the USA, more than a three-fold increase between 2007 and 2015 [10]. By June 2017, the Middle East possessed 38 da Vinci Surgical Systems, representing only 1% of those installed worldwide, and 3.2% of those installed outside the USA (Table 1). Currently, there are 19 installed da Vinci Surgical Systems in the KSA; six in Qatar; three in the UAE; two in each of Kuwait and Lebanon; and only one in Egypt.

**Table 1.** Worldwide distribution of da Vinci Surgical Systems.

Location		September 2015, *n* (%)	
North America	USA	2344 (67.4)	2369 (68.1)
	Canada	25 (0.7)	
Europe	France	90 (2.6)	586 (16.9)
	Italy	84 (2.4)	
	Germany	77 (2.2)	
	UK	55 (1.6)	
	Turkey	34 (1.0)	
	Belgium	34 (1.0)	
	Others	212 (6.1)	
Asia*	Japan	215 (6.2)	398 (11.4)
	South Korea	53 (1.5)	
	China	46 (1.3)	
	India	35 (1.0)	
	Others	49 (1.4)	
Lain America	Brazil	16 (0.5)	47 (1.4)
	Mexico	7 (0.2)	
	Argentina	6 (0.2)	
	Others	18 (0.5)	
Middle East*	Saudi Arabia	14 (0.4)	38 (1.0)
	Qatar	6 (0.2)	
	Kuwait	2 (0.1)	
	Others	16 (0.5)	
Australia/New Zealand*			36 (1.0)
South Africa			4 (0.1)
Total**			3477 (100)

*Asia lacks the addition of Asian Middle East countries and New Zealand, which were not individualised in the report **By September 2017, there were 4271 da Vinci Surgical Systems installed worldwide, including 2770 (65%) in USA, 719 (17%) in Europe, 561 (13%) in Asia, and 221 (5%) in the rest of the world. Whilst the installed base grew 13% (year-on-year) in the first quarter of 2018 to 4528 units, this number is expected to be much higher at 4806 by the end of 2018.

**Figure 1.**
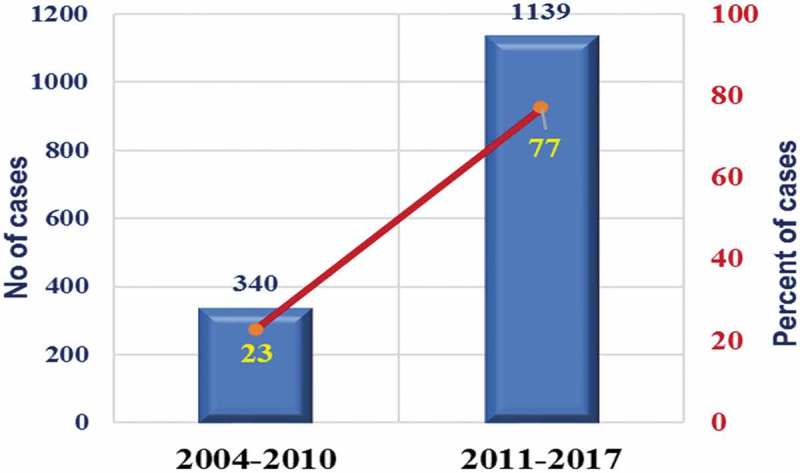
Urological RAS performed at KSA before and after 2010.

**Figure 2.**
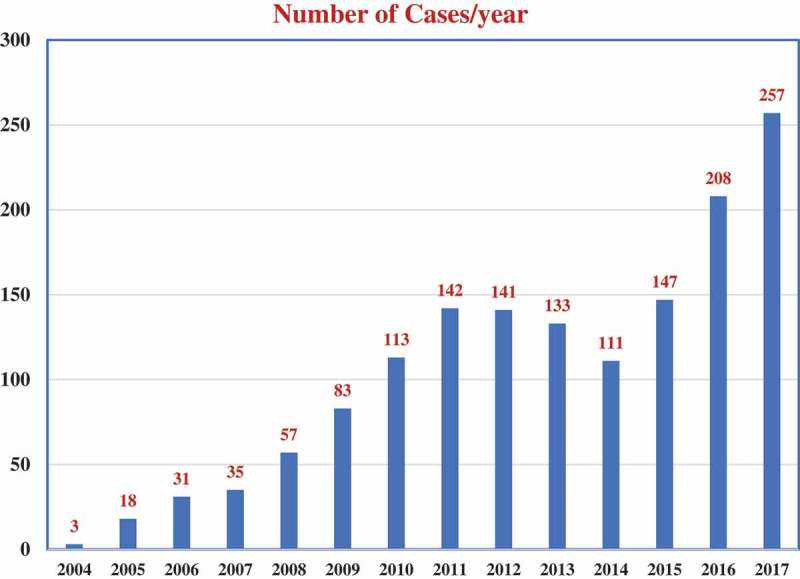
Distribution of urological RAS over years since the introduction of the *da Vinci®* robots at KSA.

The total number of RAS in the Middle East is still low compared to Europe and the USA. A total of 930 overall documented RAS procedures were performed in the KSA until December 2010, including 339 in urology, 231 in gynaecology, 209 in general surgery, 87 in paediatrics, and 46 in cardiac surgery [11]. Only 278 (29.9%) urological procedures were categorised, including 155 (55.7%) pyeloplasties, 71 (25.5%) nephrectomies, 23 (8.3%) partial nephrectomies (PNs), and 29 (10.4%) prostatectomies [11]. There was a dramatic change in RAS after 2010 until 2017, where 1139 new urological cases were performed, representing 76% of the 1497 procedures performed in the KSA; a 3.4-times increase in caseload (Figures 1 and 2). Case diversity was improved as well, from radical prostatectomy (RP), nephroureterectomy, PN, pyeloplasty, nephrectomy and pyelolithotomy before 2010 to include also ureteric reimplementation, fistula repair, pelvic lymph node dissection, ureterolysis, diverticulectomy, and radical cystectomy (RC) with intracorporeal urinary diversions (ileal conduits and neobladder). However, only a few case reports came from the KSA, and the previously described figures have been acquired from the operational records collected by the local distributor of the da Vinci Surgical System. Starting from January 2008, a total of 101 consecutive cases of robot-assisted PN (RAPN) have been performed at King Abdualziz University, KSA, and most of them were performed after 2016. During 2018, an additional 30 RAPNs have been reported within a 2-month period in two different academic centres in the KSA, as per the local distributor.

The first reported series of robot-assisted RC (RARC) and urinary diversion in the management of bladder cancer in the Middle East came from Egypt in 2004 [12]. A case series of port site metastases after RARC for muscle-invasive bladder cancer was reported 1-year later [13], and the third Egyptian report came from anaesthesiology in 2009, which highlighted the advantages of total intravenous anaesthesia during RARC [14].

**Figure 3.**
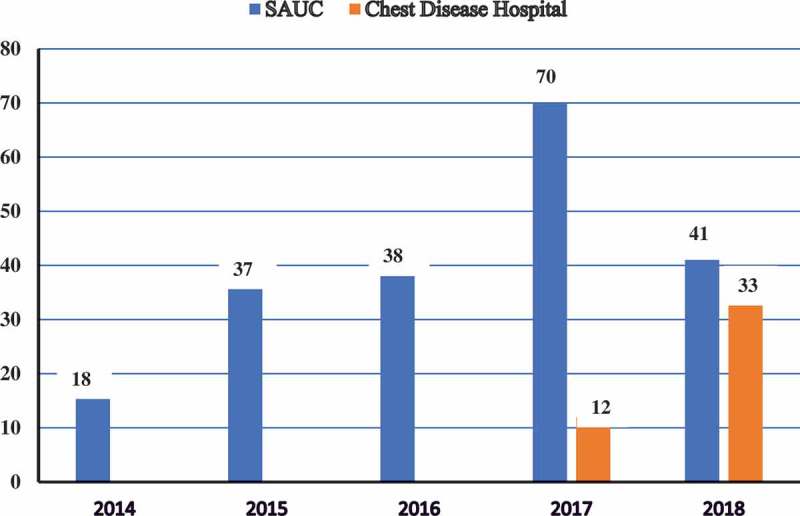
Distribution of RAS over the years since the introduction of the *da Vinci®* robots in Kuwait.

Qatar has six da Vinci Surgical Systems and established a robotic training centre for doctors and nurses on RAS and MIS. Only 183 RAS cases have been documented between 2008 and 2012, including 69% urological cases. Robot-assisted RP (RARP) was performed in 23%, pyeloplasty in 29%, PN in 21%, nephrectomy in 7%, adrenalectomy in 4%, whilst other procedures constituted ~16% [15]. The length of postoperative hospital stay and perioperative complications were comparable to open surgery. Surprisingly, these data were described later in 2016, with no data available thereafter. Kuwait has two da Vinci Si Surgical Systems, one is located at Sabah Al-Ahmad Urology Center (SAUC), a tertiary urology referral centre, and is used exclusively to perform urological procedures; and the other has been located at the Chest Disease Hospital since 2017 and it is used by thoracic and cardiac surgeons. A total of 249 RASs were performed in Kuwait until June 2018, with 204 cases performed at the SAUC, and 45 performed at the Chest Disease Hospital. Different types of urological RAS were performed between February 2014 and June 2016 including: RARP, robot-assisted radical nephrectomy (RARN), RAPN, robot-assisted pyeloplasty (RAP), robot-assisted adrenalectomy (RAA), and pyelolithotomies that failed ureteroscopic management [16–19] (Figure 3). All procedures were performed transperitoneally, and no case required open conversion or received a blood transfusion, with a median hospital stay of 3 days and no major complications (Figure 3). Lebanon has two da Vinci Surgical Systems. The American University of Beirut Medical Center performed its first RAS in July 2013. Using the newly refined da Vinci Si Surgical System, RARP was performed in 42 patients within the subsequent year and was associated with increasing the number of RPs performed by 8%, whilst decreasing the open approach by ~12% [20].

In the UAE, RAS is starting to find its place in surgical practice. Three da Vinci Si Surgical Systems are available in the UAE, two are in Abu Dhabi, and another system is located at Al-Qassimi Hospital in Sharjah, with the support of robotic proctors from the USA and neighbouring countries in the Gulf region.

## Discussion

### Overview of the da Vinci surgical robot in urology

Over the past three decades, minimally invasive robotic technology has been developed in urological practice, replacing many open urological procedures, and has substantially improved in recent years to be incorporated into everyday clinical practice. RARP was the earliest to be performed and currently, it is the most common urological RAS to date. The overall complication rate is 5–7% of Clavien Grade I–II and 4% of Clavien Grade III–IV complications [21], with a rare mortality rate of 0.1–0.2% [22]. A recent meta-analysis reported significantly lower perioperative adverse events following RARP, including re-admission rates and re-operation [23]. At 10-years postoperatively, the biochemical recurrence rate is 22.4%, with recurrence-free survival, metastasis-free survival, and cancer-specific survival rates of 73.1%, 97.5%, and 98.8%, respectively [24]. Potency recovery rates following RARP were 54–90% and 63–94%, after 12 and 24 months, respectively, which were significantly faster than open RP after 12-months [25]. Age, baseline potency status, comorbidities, and nerve-sparing procedure predict potency recovery after RARP [25].

The feasibility of RAPN has been shown, even for large and complex hilar tumours [26], with a comparable complication profile to open PN (OPN). A median warm ischaemia time of 18.8 min, estimated blood loss of 100 mL, and a positive surgical margin rate of 2.2% have been reported. Intra- and postoperative complications are 2.6% and 13%, respectively, including 3.6% high-grade Clavien III–IV complications [27,28]. The overall survival was 97% and 90%, whilst recurrence-free survival was 98.9% and 89.9% at 3 and 5 years, respectively, with 99% cancer-specific survival [29]. RAPN showed better outcomes than the laparoscopic approach in terms of warm ischaemia time, operating time, operating room time, estimated blood loss, use of haemostatic agents, and length of hospital stay [30].

RARC with lymph node dissection and urinary diversion represents a safe, effective, and technically feasible alternative to the traditional open RC (ORC). Despite the longer operative time, RARC was associated with significantly fewer complications, less blood loss, lower blood transfusion rates, shorter length of hospital stay, increased lymph node yield, and fewer positive lymph nodes than ORC, with comparable positive surgical margin rates between surgical groups [1]. A comparable re-admission rate after RARC and ORC was reported (27% vs 25.5%) [3,31]. RARC was not inferior to ORC, with a < 1% difference in 2-year progression-free survival and comparable adverse events of 67% and 69%, respectively [32]. Previous experience in RARP can decrease estimated blood loss and improve lymph node yield [33]. However, the overall complication rate following RARC is 48%, including 19% Clavien Grades III–V complications, and a 4.2% mortality rate [34]. The feasibility of robot-assisted intracorporeal urinary diversion has also been reported [35]. Dasgupta et al. [36] reported 100% overall and recurrence-free survival at 2-years, with strict adherence to oncological principles to prevent spillage of cancer cells.

Similarly, RAA is safe, feasible and effective, especially for benign adrenal disorders. There have been a few published studies on RAA using the da Vinci Surgical System [37–39]. A wide range has been reported by different centres for operative times (98–234 min), length of hospital stay (1.1–6.4 days), conversion rate for laparoscopic conversion (0–40%) and for open conversion (0–10%), and estimated blood loss (<50–576 mL) [37]. In one study, RAA was 2.3-times more costly than laparoscopic adrenalectomy, with a mean operative time of 95 min and a conversion rate of 5%. Perioperative complications were detected in 10% of cases, with no mortality recorded in the 100 operated cases [38]. However, another study reported a postoperative complication rate of 2.4%, and a mortality rate of 2.4% [39]. Length of hospital stay, complications, and conversion rates were comparable between RAA and laparoscopic adrenalectomy [40].

Academic and experienced centres in RAS have extended the clinical application to other urological procedures (Table 2). Long-term outcomes will define the role of robots in these surgeries and other emerging techniques.

**Table 2.** Status of urological RAS.

**Prostate**
Radical prostatectomy
Simple prostatectomy
**Kidney/adrenal**
Partial nephrectomy
Radical nephrectomy
Pyeloplasty
Nephroureterectomy with or without excision of bladder cuff
Extended pyelolithotomy (staghorn or multiple stones)
Renal cyst decortication/excision
Donor nephrectomy
Nephropexy
Management of chyluria
Adrenalectomy
**Ureter**
Ureteroneocystostomy
Ureteroureterostomy
Ureterectomy and re-implantation
Ureterolithotomy and ureterolysis
Ureterolympholysis
Ureteric stump excision
Ureterosciatic hernia repair
Ureteropyelostomy and ureterocalicostomy
**Bladder**
Radical cystectomy with intra/extracorporeal urinary diversion
Partial cystectomy
Diverticulectomy
Anterior pelvic exenteration
**Female urology**
Vesico-vaginal fistula repair
Vesico-uterine fistula repair
Uretero-vaginal fistula repair
Sacrocolpopexy
Bladder neck suspension

### Urological RAS in the Middle East

The KSA represents a good model for the description of RAS in the Middle East, where the first robot was introduced in 2003 [41]. The available surgical robots put the KSA at the top of the list of owners of surgical robots in the Middle East. However, these robots do not seem to have been used at a suitable rate, as reflected by the sparse number of operated cases and outgoing publications. These very few indexed cases do not fit with the many robotic systems available in the KSA. Such a small caseload may impact surgeon’s experience, resident’s training, and efficiency of RAS. However, demographic issues, referral patterns, and the prevalence of specific diseases in the KSA may also be implicated. RARP, the procedure most widely performed globally, where the magnitude of prostate cancer is very high, is significantly different from the Middle East [42]. It should be mentioned that all these robotic systems were installed in governmental hospitals, limiting the advertising and awareness of these services. A major shift in terms of robotic caseload is expected by acquiring surgical robots in private hospitals, as well.

Nevertheless, over the last 3 years, the number of returning certified fellowship-trained robotic surgeons to their home country has significantly increased, with a consequent significant increase in RAS. This is supported by the progressively increased number of RAPN performed within the last 2 years. Increased awareness, acceptance of the modern technology, and increased referral patterns amongst urologists may also play a role.

Professor Menon from Cleveland, OH, USA visited the Urology and Nephrology Center in Egypt, in 2004 with the support of Intuitive Surgical. The da Vinci Surgical System was installed only for that purpose, where 17 cases of RARC were performed. Another Egyptian report highlighted the advantages of total intravenous anaesthesia during RARC in 15 patients in 2009, by shortening the duration of pneumoperitoneum without an increase in prothrombin and fibrinogen concentrations [14]. Currently, only one da Vinci Surgical System is installed in Egypt at the National Institute of Oncology, where re-introduction of RAS is being re-evaluated, especially with the availability of expertise, scientific approach, and volume of caseload.

In Qatar, the robotic training centre shares different resources and staff with foreign centres to organise live clinical cases and experimental animal laboratory surgery. Surprisingly, these data were described later in 2016, with no data available thereafter. Despite the availability of six da Vinci Surgical Systems, RAS in Qatar may be limited by the apprehension of patients and surgeons with modern technology used to perform the surgical procedures. Moreover, a small population with a young median age in Qatar may limit the caseload. RARP was the most performed RAS procedure in Lebanon, but it seems that the prohibitive cost of the procedure, lower referral pattern, and patients’ aversion to a novel approach influenced the dwindling number of RARP compared to international figures. This is supported in part by the fact that 37% of patients underwent open RP in the robotic era, despite being aware of the modern technology [43].

### Future trends in RAS

Intuitive Surgical, developer of the da Vinci Surgical System, is currently the only manufacturer in robotic surgery. By 2019, the patents for the first generation da Vinci platform will expire, inviting new manufacturers to join the market of surgical robots [44]. Consequently, various console-based robots are expected to be developed within the next 5 years with lower costs.

Realistic near future advances in robotic systems are associated with trends toward miniature devices, micro-robots, and eventually nano-robots. Prototypes of micro-robot cameras (15 mm/3 inches) were experimentally used during laparoscopic RP and nephrectomy, giving 360° views of the surgical field. Next-generation devices are supposed to facilitate surgery and improve its outcomes, including ultra-high-definition 3D video technology, and an open console for better contact with the surgeon and the supporting staff [45]. Furthermore, distinct features may be used such as haptic gloves or cellular image guidance [44]. The haptic gloves and improved visualisation will overcome the lack of tactile feedback, which represents a main limitation of the current robotic platform.

### Future of RAS in the Middle East

RAS should not be seen as unique anymore, but as a well-established technology to achieve complex procedures easily, safely, and more efficiently. Moreover, efforts should be directed to increase public awareness in the Middle East region of this innovative technology and enhance the connection with community physicians to improve their referral patterns. Furthermore, consideration should be given to developing a training centre, with the development of a guidance committee to supervise and monitor the training and credentialing processes and outcomes. Considering that the adoption of a modern technology depends on training during residency, MIS needs to be accomplished by expanding training facilities and adding expertise to those whom can perform and are willingly to teach such procedures. Considering that many robotic surgeons in the Gulf countries, especially the KSA and Kuwait, have spent their urology residency and fellowship training in North America, a promising spurt in RAS is currently expected. This belief can be supported by the dramatic increase in the RAS caseload in the KSA of more than three-fold after 2010 until 2017, and the increase in RAS caseload in Kuwait in 2017, with the introduction of a second robotic system. With an increasing trend toward RAS, the availability of robotics should be coincidentally increased. This would support the value of the availability of skilled technical staff in the operating room; otherwise, robotic surgeons may revert to traditional surgery, even after successful RAS cases.

## Conclusion

RAS in Urology continues to grow in the Middle East, with increasing caseloads and diversity of operated cases. With the availability of fellowship-trained endourologists, scientific approach, and appropriate caseloads, the Middle East could surpass other countries in this modern technology. Many countries in the Middle East still lack surgical robots, despite having the expertise and high caseload, whilst others seem not to use the da Vinci Surgical System at a suitable rate, as reflected by the sparse number of operated cases and outgoing publications. Acceptance of robots by Middle East surgeons is significantly increasing, so that urologists are considering RAS to be the standard for RP, pyeloplasty, and PN [46].

## Declarations

All procedures performed in studies involving human participants were in accordance with the ethical standards of the institutional and/or national research committee and with the 1964 Declaration of Helsinki and its later amendments. This article is an online survey and does not contain any studies with human participants or animals performed by any of the authors.
